# Higher Dietary Fibre Increases the Faecal Microbiome Diversity of Golden Lion Tamarins (*Leontopithecus rosalia*)

**DOI:** 10.3390/ani15131831

**Published:** 2025-06-20

**Authors:** Caitlin Lawless, Katrina Kovacs, Manijeh Mohammadi Dehcheshmeh, Esmaeil Ebrahimie, Yohannes E. Messele, Mark Snowball, Darren J. Trott, David J. McLelland

**Affiliations:** 1 School of Animal and Veterinary Sciences, The University of Adelaide, Roseworthy, SA 5371, Australia; caitlin.lawless@student.adelaide.edu.au (C.L.); katrina.kovacs@student.adelaide.edu.au (K.K.); yohannes.messele@adelaide.edu.au (Y.E.M.); 2 Australian Centre for Antimicrobial Resistance Ecology, School of Animal and Veterinary Sciences, The University of Adelaide, Roseworthy, SA 5371, Australia; manijeh.mohammadidehcheshmeh@adelaide.edu.au (M.M.D.); darren.trott@adelaide.edu.au (D.J.T.); 3 Genomics Research Platform, School of Agriculture, Biomedicine and Environment, La Trobe University, Melbourne, VIC 3086, Australia; 4 Zoos South Australia, Adelaide Zoo, Frome Road, Adelaide, SA 5000, Australia; msnowball@zoossa.com.au

**Keywords:** dietary fibre, faecal microbiome, golden lion tamarin, *Leontopithecus rosalia*, microbial diversity

## Abstract

**Simple Summary:**

The bacteria living in the gut help animals extract energy from their food, synthesise nutrients, and support the proper functioning of the immune system. Gastrointestinal problems are common in Callitrichidae, making it important to understand the factors that influence their gut microbial communities. In this study, we used 16S rRNA gene sequencing to evaluate the faecal microbiome of zoo-housed golden lion tamarins (*Leontopithecus rosalia*). We observed significant compositional changes in the microbiota following an increase in dietary fibre. Notably, a beneficial genus of bacteria called *Eisenbergiella* emerged after the dietary intervention. This bacterium produces butyrate, a short-chain fatty acid that supports gut health and enhances the host ability to harvest energy from food. Following an increase in dietary fibre, the abundance of Desulfobacterota, a bacterial group associated with harmful inflammation, was reduced, suggesting an improvement in gut health. Additionally, the increase in dietary fibre led to a significant rise in bacterial diversity, which is generally considered a positive indicator of gut health.

**Abstract:**

Gut microbiota influences host energetics, metabolic rate, and overall health. Optimising the diet, such as by increasing dietary fibre, is a key strategy for promoting a healthy microbiome and improving host energy balance. In this study, we compared the faecal microbiome of five zoo-housed golden lion tamarins (*Leontopithecus rosalia*) before and after a dietary fibre increase using 16S rRNA gene sequencing. *Prevotella*, the most abundant genus, declined significantly (FDR-corrected *p* < 0.05) following the introduction of a higher-fibre diet. The dietary change also significantly altered the overall gut microbial composition, including the emergence of *Eisenbergiella* (FDR-adjusted *p* < 0.05), a butyrate-producing genus whose relative abundance increased from 0% to 0.005% (FDR-adjusted *p* < 0.05). Given the role of *Eisenbergiella* in butyrate synthesis, this shift may enhance host energy metabolism and microbial interactions. Additionally, both alpha and beta diversity increased significantly (*p* < 0.05) after the dietary fibre intervention. A significant reduction in Desulfobacterota (FDR-adjusted *p* < 0.05) following dietary fibre enrichment was observed, suggesting a shift away from microbial groups that may be associated with pathogenicity or pro-inflammatory effects. Collectively, these changes represent a positive shift in the microbiome, supporting improved host energetics and metabolic health.

## 1. Introduction

Callitrichidae (marmosets and tamarins) are commonly maintained under human care. Gastrointestinal problems, including poor faecal consistency, colitis, and ”marmoset wasting syndrome” (MWS), are common health concerns in callitrichids kept in captivity [1]. Characterised by general weakness, chronic diarrhoea, and chronic lymphocytic enteritis, MWS is considered multifactorial but remains incompletely understood [1]. The histopathology of chronic colitis in cotton-top tamarins (*Saguinus oedipus*) includes crypt abscesses, mononuclear to mixed inflammatory infiltrates in the lamina propria, and mucosal ulceration [2], and an association between colitis and colonic adenocarcinoma has been identified [2]. These lesions are comparable to those seen with chronic colitis in humans [2,3].

The natural diets of non-human primates are typically higher in dietary fibre, lower in highly digestible carbohydrate, and more diverse than diets that have been provided in human care [4,5]. Reported benefits associated with increased dietary fibre in non-human primates include improved gastrointestinal and dental health, increased natural behaviours, reduced abnormal behaviours (e.g., regurgitation in great apes; aggression and self-directed behaviour in lemurs), and a reduced risk of obesity and diabetes [1,4,6,7,8]

Lower dietary fibre and chronic stress have been proposed as two of the most important risk factors for MWS in callitrichids [1,9]. Pied tamarins (*Saguinus bicolor*) with MWS were found to have altered microbiomes compared to healthy conspecifics [9]; affected individuals had a higher relative abundance of *Lactobacillus* and *Helicobacter* and lower abundance of certain *Lachnospiraceae* and *Ruminococcaceae*, with parallels to changes seen in chronic inflammatory gastrointestinal diseases in humans. An increased abundance of *Ruminococcaceae* has been associated with higher dietary fibre in wild black howler monkeys (*Alouatta pigra*) [10].

This study evaluated the effect of a simple increase in dietary fibre on the faecal microbiome of captive golden lion tamarins (GLTs; *Leontopithecus rosalia*). A better understanding of interventions that can positively influence the gut microbiome will aid in informing management strategies that optimise the health and welfare of GLTs maintained under human care. 

## 2. Materials and Methods

### 2.1. Study Group and Ethics Statement

We examined a family group of five GLTs (a breeding pair aged 7 yr and two male and one female offspring aged 1–2 yr) maintained at Adelaide Zoo, South Australia. Each animal was individually identifiable by physical characteristics and a microchip. GLTs were housed in a facility with an indoor holding area and an outdoor aviary with access to 50 m of elevated mesh tunnels that extended over visitor pathways and garden beds. Direct sunlight was accessible for large parts of the day in outdoor areas.

Formal Animal Ethics Committee approval was not required for this study. This study opportunistically evaluated the effect of a planned diet change implemented by the zoo nutritionist at Zoos South Australia, Adelaide, Australia. The dietary adjustment (increased fibre via cellulose and psyllium in the primate cake) was part of routine animal management and not introduced for the purpose of experimental intervention. Faecal samples were collected non-invasively, either from enclosure surfaces shortly after defaecation in publicly accessible areas or during standard enclosure maintenance by zookeepers. There was no handling, intervention, or disturbance to the animals beyond routine care procedures. Given the observational and non-invasive nature of this research, Animal Ethics Committee approval was not required. However, the project was reviewed and approved by the Research Approval Committee at Zoos South Australia, which oversees research activity within the institution.

### 2.2. Diet Composition

This study opportunistically evaluated the effect of a planned diet change to increase the fibre content of the GLT diet. The increase in dietary fibre was achieved by adding cellulose and psyllium to the ”primate cake” portion of the diet, while all other dietary components remained unchanged (Table 1). The calculated nutritional compositions of the two primate cake recipes, as well as the overall compositions of Diets 1 and 2, are outlined in Table 2. Diet 1 had been used for approximately five years prior to the dietary change.

### 2.3. Sample Collection

Faecal samples, individually identified, were collected directly into zip-lock plastic bags from the enclosure or beneath elevated mesh tunnels. Following collection, samples were placed on ice within 15 min of defaecation and transferred to a −80 °C freezer within 30 min of collection. Sample collection for Diet 1 spanned an 8-week period. For Diet 2, a 3-week sample collection phase commenced three weeks after the introduction of the fibre-enhanced diet to allow for dietary acclimation.

A negative (blank) control sample was included throughout the DNA extraction and sequencing workflow to monitor potential contamination from reagents, air, water, and the laboratory environment.

### 2.4. DNA Extraction

DNA was isolated from faecal samples using the QIAamp DNA Stool Kit (QIAGEN, Hilden, Germany), following the manufacturer’s instructions with modifications. Frozen samples were weighed, and 180–220 mg aliquots were transferred to 2 mL microcentrifuge tubes. Then, 1.4 mL of Buffer ASL was added to each tube, followed by vortexing for 1 min. To ensure complete emulsification—critical due to the kit’s original design for human faecal samples—vortexing duration was extended beyond the protocol’s recommendations. The suspension was incubated at 90 °C for 5 min with the lids removed to prevent pressure-related contamination then vortexed for 15 s. Samples were centrifuged at 14,000× *g* for 1 min; 1.2 mL of supernatant was transferred to a fresh 2 mL microcentrifuge tube; and the pellet was discarded. One InhibitEX tablet was added to each sample and vortexed for 3 min. The suspension was incubated at room temperature for 1 min, after which the supernatant was transferred to 1.5 mL microcentrifuge tubes and centrifuged at 14,000× *g* for 5 min. The entire supernatant was pipetted into a fresh 1.5 mL microcentrifuge tube, and the pellet was discarded. Finally, the supernatant was centrifuged again for 3 min, and 200 μL of the resulting supernatant was combined with 15 μL of Proteinase K in a new 1.5 mL microcentrifuge tube.

Four microlitres (4 μL) of RNase was added to degrade potential environmental RNA contaminants. Subsequently, 200 μL of Buffer AL was added to the mixture and vortexed for 15 s. Samples were incubated at 70 °C for 10 min, followed by the addition of 200 μL of ethanol to the lysate and brief vortexing. The lysate was then transferred to a fresh QIAamp spin column seated in a new collection tube and centrifuged at 14,000× *g* for 1 min. The spin column was moved to a clean collection tube, and 500 μL of Buffer AW1 was added before centrifugation (14,000× *g*, 1 min). The collection tube was replaced, 500 μL of Buffer AW2 was added, and the column was centrifuged again at 14,000× *g* for 3 min. This Buffer AW2 step was repeated twice more to enhance DNA purity. Finally, the spin column was transferred to a sterile 1.5 mL microcentrifuge tube, 200 μL of Buffer AE was added, and the mixture was incubated at room temperature for 1 min before a final centrifugation (14,000× *g*, 1 min) to elute the DNA.

DNA quality was assessed using a NanoDrop 1000 spectrophotometer (Thermo Scientific, Waltham, MA, USA), with absorbance ratios (260/280 and 260/230) evaluated to confirm purity. The extracted DNA was amplified by PCR using primers targeting the 550 bp V3–V4 region of the bacterial 16S rRNA gene to verify bacterial DNA presence as a quality test [12]. Once the presence of bacterial DNA was confirmed by PCR, the samples were submitted for sequencing.

### 2.5. High-Throughput Sequencing of the 16S rRNA Gene V3–V4 Hypervariable Regions Using the Illumina MiSeq Platform

The 550 bp V3–V4 hypervariable region of the bacterial 16S rRNA gene was amplified using published primers. PCR products were visualised by electrophoresis on agarose gels followed by Gel Red™ staining to confirm the presence of amplified DNA in each sample (~550 bp bands). Library preparation and sequencing for 16S microbiota analysis were performed at the South Australian Genomics Centre, South Australian Health & Medical Research Institute DNA Sequencing Facility (SAHMRI). The Illumina library preparation protocol was followed, incorporating the following primers: forward (CCTACGGGNGGCWGCAG) and reverse (GACTACHVGGGTATCTAATCC). Amplicon sequencing was carried out on the Illumina MiSeq platform using V3 SBS chemistry (Illumina, Inc., San Diego, CA, USA), generating paired-end reads of 300 bp each (2 × 300 bp).

### 2.6. Microbiota Profiling

To obtain high-quality sequencing results for microbiota profiling and comparative analysis, raw reads underwent several preprocessing steps, including adapter trimming, fixed-length filtering, and merging of paired-end reads. Samples with low coverage were excluded using read-count-based filtering.

The taxonomic assignment of reads from each sample was carried out using the CLC Microbial Genomics Module of CLC Genomics Workbench (QIAGEN, version 25), as previously described [12,13]. Operational taxonomic units (OTUs), which represent clusters of sequences with high similarity, were used as proxies for microbial taxa. OTU analysis was performed at 99% sequence similarity. Representative sequences (cluster centroids) were generated for each OTU. The SILVA database (version 138.1) was used for 16S rRNA gene referencing [14,15,16], and OTU abundances were quantified based on read counts.

### 2.7. Statistical Analysis

In this study, we used median instead of mean for more reliable interpretations of microbiome data. Due to the common non-normal distribution of microbial abundance data, high variability, the presence of outliers, zero inflation, and skewness, the median is preferred over the mean as a measure of central tendency in microbiome studies [17,18]. Unlike the mean, which is heavily influenced by extreme values and assumes symmetry, the median provides a robust estimate of central tendency that better represents typical microbial abundances in skewed distributions. This is particularly critical in compositional microbiome data, where relative abundances sum to 1, and changes in one taxon inherently affect all others. The median is resistant to outliers and ensures stable estimates even when rare taxa dominate individual samples.

Microbiome count data were analysed for differentially abundant taxa using a negative binomial generalized linear model (GLM) implemented in the DESeq2 package (Version 1.47.5 in the R environment). Raw counts were normalised internally by DESeq2’s median-of-ratios method to correct for uneven sequencing depth. Statistical significance was assessed via the Wald test, with taxa exhibiting a Benjamini–Hochberg-adjusted false discovery rate (FDR) < 0.05 classified as differentially abundant between comparator groups (Diet2 vs. Diet 1).

Differential abundance analysis was performed at the phylum and genus taxonomic levels. Sub-genus classifications (e.g., *Prevotella_9*, *Bacteroides_2*) were merged with their parent genera (*Prevotella*, *Bacteroides*) to align with formal taxonomic hierarchies and ensure consistency in genus-level analyses. This conservative approach mitigates the overinterpretation of database-specific clade labels (e.g., SILVA v138 or Greengenes 13_8 designations), which lack validated species or functional distinctions in the absence of supporting genomic or phenotypic evidence.

DESeq2 was prioritised over alternatives (e.g., edgeR, LEfSe) based on benchmarking studies, demonstrating its superior precision–recall balance in 38 microbiome datasets [19]. The tool’s robust handling of overdispersion through shrinkage estimation and its capacity to model sparse, zero-inflated count data align with typical microbiome dataset characteristics [20]. All analyses excluded taxa with fewer than 10 total counts across samples to reduce false positives.

Alpha diversity was used for estimating the diversity within samples, based on the Shannon Index. The Kruskal–Wallis H test was applied to test the statistical significance of alpha diversity between the two diets. Kruskal–Wallis H evaluates whether the values of two diets come from the same distribution or originate from different diets. A significant *p*-value indicates that the diets have different distributions.

Beta diversity can be used to evaluate the change in diversity between two groups. Beta diversity was calculated by estimating the distance between each pair of samples and then by performing Principal Coordinate Analysis (PCoA) on the distance matrices using Bray–Curtis distance matrices, as previously described [12]. The PERMANOVA test was also employed to calculate the effect of size and significance on beta diversity in comparisons of the two diets. PERMANOVA obtains its significance from a permutation test. The number of permutations was set to 99,999. The abovementioned analyses were performed using the Microbiome plugin of CLC Microbial Genomics Workbench (QIAGEN).

## 3. Results

### 3.1. Sequenced Faecal Samples from GLTs

A total of 45 faecal samples that yielded high-quality DNA and met the quality and quantity criteria were selected for sequencing, along with one negative control sample (n = 46 in total). Of the faecal samples, 20 were from animals fed Diet 1, and 25 were from those fed Diet 2 (higher dietary fibre), as detailed in Table 3.

### 3.2. GLT Microbiota

**Figure 1:**
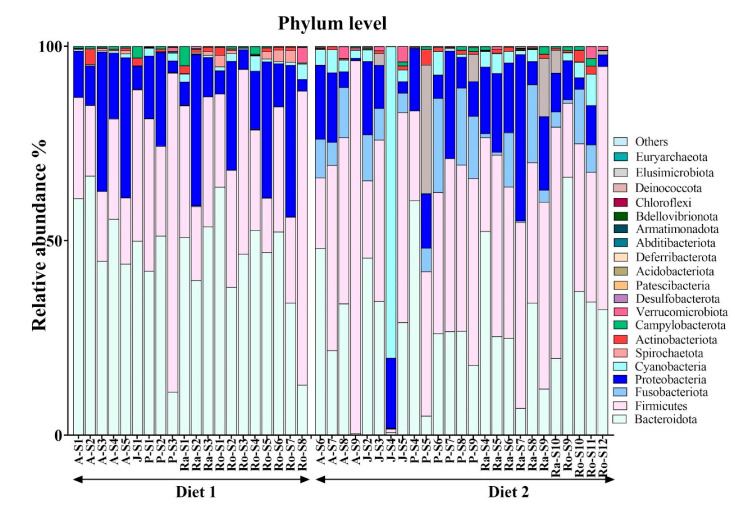
Relative abundance at the phylum level of the faecal microbiome of five golden lion tamarins (*Leontopithecus rosalia*) before (Diet 1, n = 20) and after (Diet 2, n = 25) an increase in dietary fibre.

**Figure 2:**
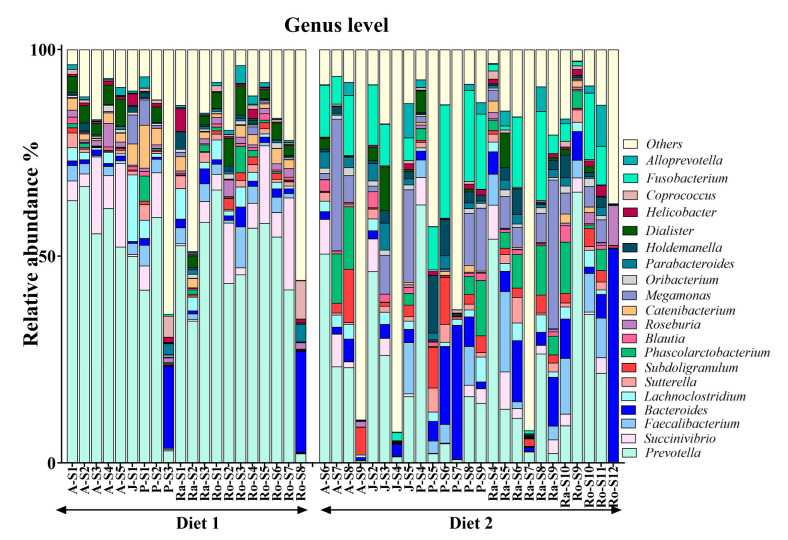
Genus-level comparison of faecal microbiome relative abundance in five golden lion tamarins (*Leontopithecus rosalia*) before (Diet 1, n = 20) and after (Diet 2, n = 25) an increase in dietary fibre.

Bacteroidota, Firmicutes, and Proteobacteria were the dominant phyla in the GLT gut microbiota, with median relative abundances of 0.37, 0.36, and 0.12, respectively. The three mentioned phyla collectively accounted for approximately 85% of the microbial community (Figure 1). At the genus level, *Prevotella*, *Succinivibrio*, *Faecalibacterium*, and *Bacteroides* were the most prevalent genera (Figure 2), with *Prevotella* being the most abundant genus, exhibiting a median relative abundance of 0.35.

The negative (blank) control sample yielded a low number of reads (3878), indicating minimal contamination from reagents, water, or ambient air. The low read count in the negative control provides confidence that significant background bacterial contamination was absent. The inclusion of negative controls throughout the workflow validates the integrity of the sequencing results and ensures that the observed microbial profiles accurately reflect the GLT microbiota, rather than being confounded by environmental or laboratory-derived contaminants.

### 3.3. Phylum-Level Microbial Shifts in Response to High-Fibre Diet

The high-fibre diet (Diet 2) led to notable shifts in the faecal microbiota of GLTs (Table 4). Fusobacteriota showed a substantial increase in median relative abundance from 0.000357 to 0.06 (FDR-corrected *p* < 2.4 × 10^−29^). Other phyla that significantly increased included Acidobacteriota and Cyanobacteria, with FDR-corrected *p*-values of 0.0011 and 0.0012, respectively. Conversely, Spirochaetota and Desulfobacterota exhibited significant reductions in abundance, with corrected *p*-values of 4.49 × 10^−13^ and 3.20 × 10^−4^, respectively. These changes suggest that dietary fibre intake can selectively enrich or suppress specific microbial phyla in tamarin gut microbiota.

### 3.4. Alterations in Genus-Level Gut Microbiota After Higher-Fibre Dietary Intervention

Significant differences in bacterial genus abundance were observed in faecal samples after an increase in dietary fibre (Table 5). Specifically, the relative abundances of *Prevotella*, *Succinivibrio*, *Dialister*, *Catenibacterium*, and *Megasphaera* were all significantly reduced following the higher-fibre diet (Diet 2). For instance, *Prevotella* exhibited a substantial decrease from a median relative abundance of 0.536 to 0.160 (FDR-corrected *p* = 1.99 × 10^−5^), while *Catenibacterium* and *Megasphaera* also showed marked reductions (FDR-corrected *p* = 2.41 × 10^−6^ and 5.24 × 10^−7^, respectively).

As presented in Table 5, the transition to a high-fibre diet correlated with the emergence of *Psychrobacter*, *Variovorax*, *Comamonas*, *Mucispirillum*, *Duganella*, *Hymenobacter*, *Odoribacter*, *Eisenbergiella*, *Chryseobacterium*, and *Cardiobacterium*. *Eisenbergiella* abundance in gut microbiota increased from a median relative abundance of 0% to 0.005% (FDR-corrected *p* = 2.72 × 10^−14^). The median value of relative abundance of several genera such as *Phascolarctobacterium*, *Fusobacterium*, *Solobacterium*, and *Subdoligranulum* showed a significant increase (FDR-corrected *p* < 0.05) (Table 5).

### 3.5. High-Fibre Diet Increased Alpha Diversity in Tamarin Gut Microbiota

**Figure 3:**
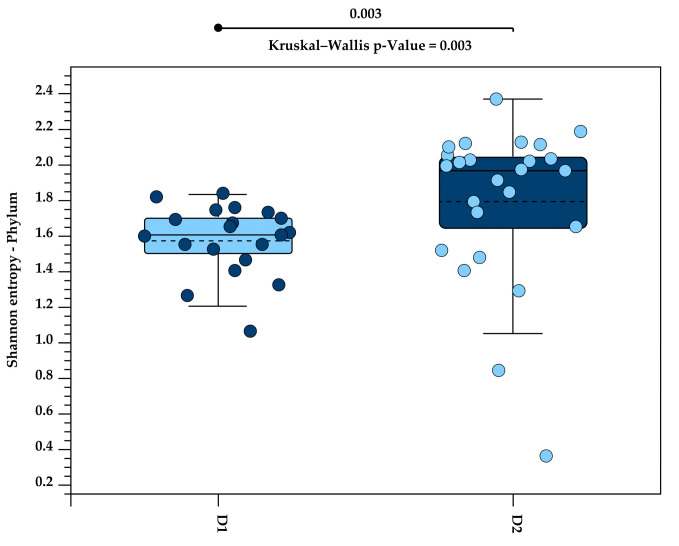
Alpha diversity at the phylum level, measured using the Shannon Index, in the faecal microbiota of five golden lion tamarins (*Leontopithecus rosalia*) before (Diet 1/D1, n = 20) and after (Diet 2/D2, n = 25) an increase in dietary fibre. Each circle represents an individual sample. The solid line within each box indicates the median, while the dashed line indicates the mean Shannon entropy for each group. Box colours distinguish between Diet 1 and Diet 2 groups. A significant increase in alpha diversity was observed following the high-fibre diet (Kruskal–Wallis *p* = 0.003).

**Figure 4:**
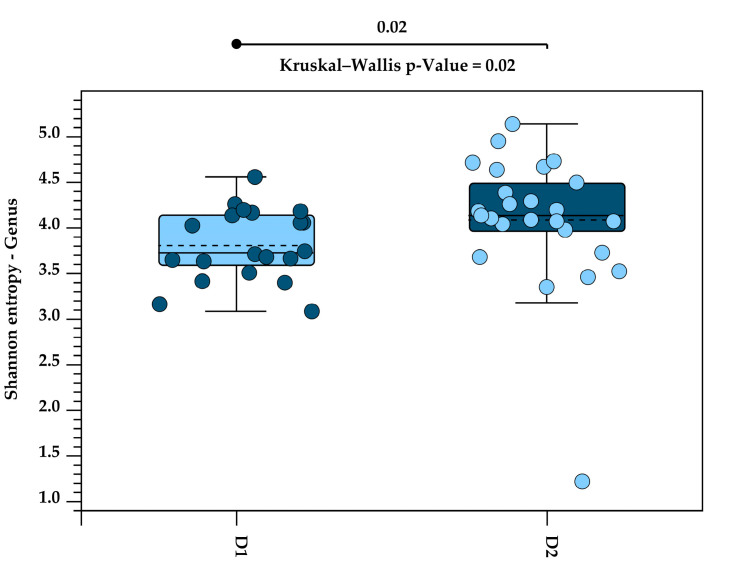
Alpha diversity at the genus level, measured using the Shannon Index, in the faecal microbiota of five golden lion tamarins (*Leontopithecus rosalia*) before (Diet 1, n = 20) and after (Diet 2, n = 25) an increase in dietary fibre. Individual samples are shown as circles. The median Shannon entropy for each group is indicated by the solid line within each box, and the mean by the dashed line. Box colors differentiate between Diet 1 and Diet 2. A significant increase in alpha diversity was observed following the higher-fibre diet (Kruskal–Wallis *p* = 0.02).

A significant increase in alpha diversity was observed with the higher fibre content in Diet 2, compared to Diet 1, at both the phylum level (*p* = 0.003; Figure 3) and the genus level (*p* = 0.02; Figure 4).

### 3.6. Beta Diversity Enhanced Following Increased Dietary Fibre Intake

**Figure 5:**
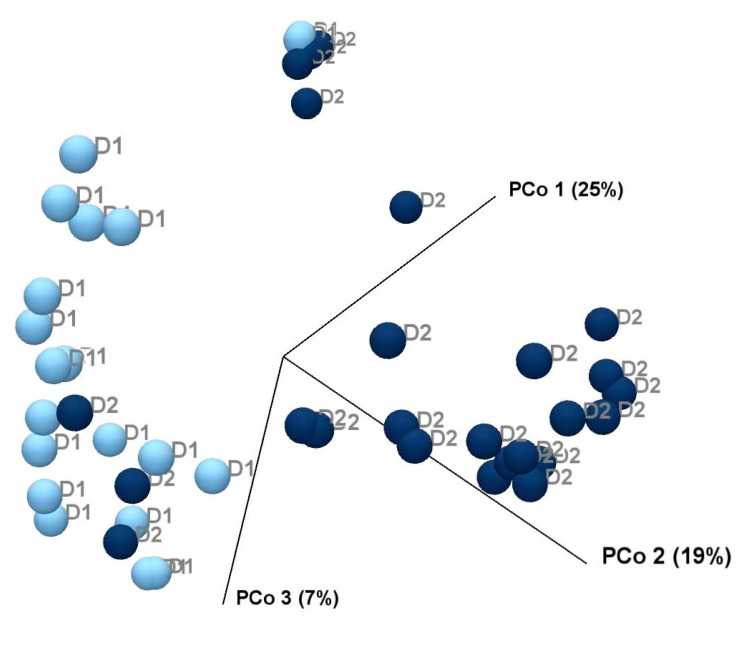
Principal Coordinate Analysis (PCoA) plot based on Bray–Curtis dissimilarity showing the faecal microbiome composition of five golden lion tamarins (*Leontopithecus rosalia*) before (Diet 1/D1, n = 20) and after (Diet 2/D2, n = 25) an increase in dietary fibre. Light blue dots represent Diet 1 samples, and dark blue dots represent Diet 2 (higher-fibre) samples.

The microbial community composition (beta diversity) differed significantly between Diet 1 and Diet 2, as shown by a Principal Coordinate Analysis (PCoA) of Bray–Curtis dissimilarity values (Figure 5). Samples from Diet 1 clustered separately from those of Diet 2, indicating a clear shift in microbial community structure following the high-fibre intervention. The first principal coordinate explained 25% of the total variance in the microbiome data, highlighting its contribution to the observed separation between the two dietary groups. PERMANOVA analysis also confirmed the significant difference in beta diversity in *p* < 0.05.

## 4. Discussion

This study employed 16S rRNA gene sequencing to first profile faecal microbiota of GLTs and then compare the faecal microbiomes of GLTs before and after an increase in dietary fibre. The findings of this study demonstrated that there were significant differences in microbial composition and diversity with the higher-fibre diet.

The increase in the fibre content of the GLT diet was achieved by adding psyllium powder and medium-length cellulose powder to the primate cake. Psyllium is an excellent source of soluble fibre and has a range of benefits for human patients with chronic gastrointestinal disease: increasing meal viscosity, delaying gastric emptying, extending colon transit time, increasing the bulk of stools, and inhibiting lactulose-induced colonic mass movements, offering improvement for both diarrhoea and constipation [21]. Cellulose is a source of insoluble and poorly fermentable fibre, with benefits for animal health [22]. In a mouse model of endotoxaemia, cellulose supplementation decreased intestinal hyperpermeability and apoptosis [23].

The dominance of Bacteroidota, Firmicutes, and Proteobacteria in GLT microbiota aligns with microbial profiles commonly observed in other primates. These findings are consistent with previous studies of the faecal microbiomes of zoo-housed GLTs and other callitrichids [24], wild saddleback tamarins (*Leontocebus weddelli*) [25], and of humans [26]. In contrast, captive and wild marmosets (*Callithrix* spp.) were reported to have microbiomes dominated by Gammaproteobacteria and Campylobacteria, respectively [27].

We identified *Prevotella* as the most abundant genus in faecal microbiota of GLTs. *Prevotella* (Bacteroidota) has previously been reported as the most abundant genus in zoo-housed callitrichids [24]. Higher relative abundances of *Prevotella* and *Bacteroides* in captive primates have been previously reported, in contrast to wild populations [28]. *Bifidobacterium* (Actinomycetota), important for carbohydrate metabolism, has been reported as the most abundant genus in wild GLTs [29]. In this study, we identified *Bifidobacterium* in low abundance in captive GLTs with a median relative abundance of 0.001 (0.1% of microbiota).

The high prevalence of *Prevotella*, along with genera such as *Succinivibrio*, *Faecalibacterium*, and *Bacteroides*, suggests a microbiome well-adapted to carbohydrate-rich diets [30]. These baseline community structures are important for interpreting how dietary changes, like increased fibre intake, can drive shifts in both microbial composition and host metabolic outcomes [31].

The decline in the relative abundance of *Prevotella* after the higher-fibre diet in this study contradicts its typical association with fibre-rich diets in humans, suggesting alternative taxa may be more dominant in this role in these GLTs. The observed enrichment of Fusobacteriota and *Fusobacterium* in response to a high-fibre diet is unusual as they are often associated with proinflammatory states in humans. However, some *Fusobacterium* species are capable of producing butyrate, a short-chain fatty acid beneficial for colon health [32], and their role in non-human primates may differ. The reduction in Desulfobacterota following fibre enrichment reflects a shift away from potentially pathogenic or pro-inflammatory microbiota [33].

The decrease in *Succinivibrio*, *Dialister*, *Catenibacterium*, and *Megasphaera* observed in our study may reflect a shift towards a gut environment less conducive to these genera, possibly due to increased fermentation of complex carbohydrates and subsequent changes in short-chain fatty acid production.

The gut microbiome plays a central role in shaping host energetics and metabolic rate [34,35]. The emergence of *Eisenbergiella* in the GLT microbiota on Diet 2 (median relative abundance of 0% → 0.005%, FDR-corrected *p* = 2.72 × 10^−14^) aligns with its known butyrate-producing capabilities, potentially enhancing host energy harvest [34,35]. *Eisenbergiella tayi* specifically produces butyrate as a major metabolic end product [36]. *Eisenbergiella* dominance in microbial communities correlates with increased butyric acid production. Butyrate’s role in energy metabolism and host–microbe interactions is well-documented, including its capacity to improve energy harvest efficiency [34,37]. These shifts suggest fibre-induced microbial specialisation, though longitudinal studies are needed to separate diet effects from confounding factors like host adaptation.

An increase in the abundance of genera within the Firmucutes was the dominant reported change to the microbiome when fruit in the diet of zoo-housed callitrichids was replaced with higher-fibre vegetables [24]. The types of Firmicutes contributing to this change varied across hosts, though *Phascolarctobacterium*, associated with the digestion of complex plant fibres, was identified as increasing in most of the species studied. Our results documented a significant increase (FDR-corrected *p* < 0.05) in the median relative abundance of *Phascolarctobacterium* following the increase in dietary fibre.

There was a significant increase in alpha and beta diversity with the increase in dietary fibre on Diet 2. It has been argued that reduced dietary fibre is associated with reduced diversity in the microbiomes of humans and captive primates [28]. Our results suggest that by increasing dietary fibre, we can go some way to reversing that trend. However, captive callitrichids had inconsistent changes in microbial diversity when fruit was replaced with higher-fibre vegetables [24]. There was no observed change in alpha diversity when captive lemurs were provided with supplemental romaine lettuce for a 10 d period, though intra-individual differences became more significant with time on the supplemented diet [25]. The characteristics of the microbiome prior to the diet change, the amount by which fibre increased, the type of fibre introduced, the period of time the microbiome was monitored, and other factors influencing the microbiome are all factors that could affect the observed changes in diversity across studies.

We assessed a single family group of five GLTs. Expanding this research to a larger number of animals, a broader range of species, and across multiple settings would be beneficial to assessing whether the microbiome changes observed here in response to increased dietary fibre can be replicated. Additionally, there is much scope to explore the relative benefits of different strategies for increasing dietary fibre and to refine dietary recommendations in general through this type of research.

Samples were collected over 8 w and 3 w for Diets 1 and 2, respectively, though were treated as point-in-time sampling for the purposes of analysis. Zoo-housed callitrichids that transitioned from fruit to higher-fibre vegetables were sampled up to 6 months prior and 12 months following the diet change, with significant microbiome changes occurring over time [24]. In the present study, sampling over a longer period may have revealed additional changes to the microbiome over time in response to the increase in dietary fibre.

The study period extended from April–May (autumn) for Diet 1 samples to July–August (Winter) for Diet 2 samples. There is the potential for season to influence the results of microbiome research [26]. A study of the caecal microbiome of broiler chickens found significantly differences across the four seasons [38]. In a cross-sectional human study, Bacteroidota were less abundant and Actinobacteria more abundant in summer, while Firmicutes were seasonally independent [26].

The limitation of this study was the use of partial 16S rRNA gene sequencing targeting only the V3-V4 regions of the bacterial 16S rRNA gene, rather than full-length 16S rRNA gene sequencing. While V3-V4-based short-read sequencing is widely used due to its cost-effectiveness and established protocols, it is less effective at resolving bacterial taxa at the species level and may lead to underestimation of microbial diversity compared to full-length 16S approaches. Recent studies have demonstrated that long-read 16S sequencing technologies, such as PacBio or Nanopore platforms, provide more comprehensive and accurate microbiome profiles, enabling species-level identification and improved detection of microbial diversity [39]. Therefore, a future direction for GLT microbiome research would be to employ long-read 16S rRNA gene sequencing to achieve more comprehensive and precise profiling of the microbiota, including robust species-level identification and improved detection of low-abundance or rare taxa.

## 5. Conclusions

Dietary fibre is a well-established modulator of gut microbial communities. In this study, the inclusion of psyllium and cellulose in the diets of zoo-housed golden lion tamarins (GLTs) led to increased faecal microbiome diversity and significant compositional shifts. Overall, these changes reflect a positive restructuring of the microbiota and better shaping host energetics and metabolic rate. Such microbial adjustments are likely to reduce the incidence of gastrointestinal problems and enhance the general health of GLTs in managed care. Notably, these benefits were observed following a relatively modest dietary modification, highlighting the potential of targeted nutritional strategies to improve the welfare and management of captive callitrichids. The high-fibre intervention promoted the enrichment of metabolically active and potentially beneficial taxa while suppressing microbial groups linked to pathogenicity and inflammation.

## Figures and Tables

**Table 1:** Golden lion tamarin (*Leontopithecus rosalia*) diet (per animal per day; five animals fed as a group) before and after an increase in dietary fibre achieved by modifying the primate cake recipe (the amounts fed remained constant).

Diet Item	Amount (g)
”Primate Cake” ^a^	100
Group A (leafy green) vegetables	11
Group B (other non-starchy) vegetables	38
Group C (starchy) vegetables	38
High protein supplement powder ^b^	5
Dry dog food ^c^	3
Discretionary item ^d^	x1 serve

^a^ Primate Cake Recipe Diet 1: Wombaroo Primate Meal 100 g (Wombaroo Food Products, Glen Osmond, South Australia), spinach/silverbeet 20 g, celery 13 g, parsley 3 g, flaxseed oil 7 mL, water/herbal tea 120 mL. Primate Cake Recipe Diet 2: Wombaroo Primate Meal 100 g, cellulose powder 18 g, psyllium powder 0.6 g, spinach/silverbeet 25 g, celery 13 g, parsley 4 g, flaxseed oil 9 mL, water/herbal tea 94 mL. ^b^ Wombaroo High Protein Supplement, Womabroo Food Products, Glen Osmond, South Australia. ^c^ ADVANCE™ Adult Dental Care Medium Breed, Mars Australia Pty Ltd., Wodonga, Victoria. ^d^ One of the following: boiled chicken meat 8 g; cheese 3 g; mealworms 5 g; cockroaches 5 g; crickets 5 g; stick insects 1 g; or hard-boiled egg 10 g.

**Table 2:** Calculated nutritional composition (dry matter basis) of the primate meal, primate cake, and the total diet (per animal) of five captive golden lion tamarins (*Leontopithecus rosalia*) before (Diet 1) and after (Diet 2) an increase in dietary fibre. Available National Research Council (NRC) recommendations [11] included for comparison.

	Primate Meal		Primate Cake		Total Diet		NRC (2003) [11]
	Diet 1	Diet 2	Diet 1	Diet 2	Diet 1	Diet 2	
Dry Matter (%)	94.0	94.1	39.2	47.1	29.9	33.3	–
Energy (Kcal/ 100 g) ^	356	299	380	333	368	342	–
Protein (%) ^	21.7	18.3	20.5	17.2	24.3	21.6	15–22
Fat (%) ^	4.1	3.5	9.4	9.8	9.7	9.9	–
Ash (%) ^	8.9	7.5	8.6	7.3	7.8	7.0	–
NDF (%)	14.9	26.9	13.6 ^1^	24.4 ^3^	9.2 ^5^	17.3 ^5^	>10.0
ADF (%)	0.0	11.8	0.0 ^1^	10.7 ^3^	0.1 ^6^	7.6 ^6^	>5.0
TDF (%) ^	–	0.5	0.8 ^2^	1.2 ^4^	4.8 ^7^	4.6 ^8^	–
NFC (%) *	50.4	43.8	47.9	41.3	49.0	44.2	–

^ no energy, protein, fat, ash, TDF, and NFC data for cellulose. * NFC, non-fibre carbohydrate, is made up of starch, simple sugars and soluble fibre, calculated as 100 − (protein + fat + ash + NDF). ^1^ Data limited to primate meal. ^2^ Data limited to spinach, celery, parsley, flaxseed oil, and water. ^3^ Data limited to primate meal and cellulose. ^4^ Data limited to spinach, celery, parsley, flaxseed oil, water, and psyllium. ^5^ Data limited to primate meal and cellulose, contained in the “primate cake”, and the insects. ^6^ Data limited to primate meal and cellulose, contained in the “primate cake”. ^7^ Data limited to spinach, celery, parsley, flaxseed oil, water, and psyllium, contained in the “primate cake”, as well as group A, B, and C vegetables; chicken; cheese; and egg). NB: no “fibre” (NDF, ADF, TDF) data available for the Wombaroo high protein supplement or ADVANCE dental dry dog food products. ^8^ Total Dietary Fibre (TDF) in the total diet analysis does not include cellulose; hence, the values are static between the diets.

**Table 3:** Summary of sequenced faecal samples from five golden lion tamarins (*Leontopithecus rosalia*) at Adelaide Zoo, yielding high-quality DNA using the QIAamp DNA Stool Kit. Quality criteria: DNA concentration > 25 ng/μL, 260/280 ratio ≈ 1.8, and 260/230 ratio > 1.8 per individual.

Animal	Sex	Age (Years)	Samples with Good Quality DNA	
			Diet 1	Diet 2
Roscoe	M	7	8	4
Juniper	F	1	1	5
Arella	F	7	5	3
Poko	M	2	3	7
Rafael	M	2	3	6
Total			20	25

**Table 4:** Differentially abundant bacterial phyla in faecal samples of five golden lion tamarins (*Leontopithecus rosalia*) before (Diet 1, n = 20) and after (Diet 2, n = 25) increased dietary fibre. Results are based on DESeq2 Wald tests with Benjamini–Hochberg FDR correction.

Phylum	Median of Relative Abundance		Direction in Higher-Fibre Diet (Diet 2)	*p*-Value	FDR-Corrected *p*-Value
	Diet 1	Diet 2			
Fusobacteriota	0.000357	0.06	Increased	0.0000	0.0000
Spirochaetota	0.001765	0.0000521	Decreased	0.0000	0.0000
Desulfobacterota	0.0003985	0.000194	Decreased	0.0000	0.0003
Acidobacteriota	0	0.00000843	Increased	0.0002	0.0011
Cyanobacteria	0.006805	0.03	Increased	0.0003	0.0012
Deferribacterota	0	0.00000815	Increased	0.0015	0.0048
Bacteroidota	0.485	0.27	Decreased	0.0017	0.0048
Campylobacterota	0.00327	0.0025	Decreased	0.0028	0.0071
Patescibacteria	0.000007145	0.0000218	Increased	0.0107	0.0245
Proteobacteria	0.135	0.11	Decreased	0.0263	0.0550

**Table 5:** Differences in the genus-level abundance of bacteria between samples taken from five golden lion tamarins (*Leontopithecus rosalia*) before (Diet 1, n = 20) and after (Diet 2, n = 25) an increase in dietary fibre.

Genus	Median of Relative Abundance		Direction in Higher-Fibre Diet (Diet 2)	*p*-Value	FDR-Corrected *p*-Value
	Diet 1	Diet 2			
*Prevotella*	0.53620032	0.160230548	Decreased	0.0000	0.0000
*Succinivibrio*	0.054766508	0.027215864	Decreased	0.0000	0.0000
*Dialister*	0.02799577	0.001178487	Decreased	0.0059	0.0242
*Catenibacterium*	0.022250012	0.001121756	Decreased	0.0000	0.0000
*Megasphaera*	0.004360297	0.002163387	Decreased	0.0000	0.0000
*Anaerovibrio*	0.002077639	0.000183251	Decreased	0.0000	0.0001
*Treponema*	0.001095639	0.0000478	Decreased	0.0000	0.0001
*Coprococcus*	0.003996526	0.002971945	Decreased	0.0004	0.0021
*Butyricicoccus*	0.00227945	0.001463972	Decreased	0.0012	0.0056
*Allisonella*	0.000600753	0.000172341	Decreased	0.0000	0.0002
*Brachyspira*	0.000220978	0	Decreased	0.0075	0.0286
*Acidaminococcus*	0.000120786	0	Decreased	0.0000	0.0000
*Mitsuokella*	0.000098648	0.00000822	Decreased	0.0000	0.0000
*Asteroleplasma*	0.00006855	0.00000822	Decreased	0.0096	0.0341
*Moryella*	0.000009275	0	Decreased	0.0042	0.0182
*Victivallis*	0.00000445	0	Decreased	0.0048	0.0199
*Faecalitalea*	0.00000389	0	Decreased	0.0101	0.0348
*Stenotrophomonas*	0.00000389	0.0000109	Increased	0.0135	0.0446
*Psychrobacter*	0	0.00000917	Increased	0.0000	0.0000
*Variovorax*	0	0.00000956	Increased	0.0000	0.0001
*Comamonas*	0	0.00000956	Increased	0.0006	0.0031
*Mucispirillum*	0	0.0000108	Increased	0.0002	0.0013
*Duganella*	0	0.0000131	Increased	0.0017	0.0077
*Hymenobacter*	0	0.0000164	Increased	0.0000	0.0001
*Leuconostoc*	0.00000389	0.0000215	Increased	0.0134	0.0446
*Odoribacter*	0	0.0000265	Increased	0.0000	0.0000
*Chryseobacterium*	0	0.000028	Increased	0.0005	0.0027
*Eisenbergiella*	0	0.000052	Increased	0.0000	0.0000
*Cardiobacterium*	0	0.0000544	Increased	0.0000	0.0002
*Fusicatenibacter*	0.000082	0.000137358	Increased	0.0000	0.0000
*Peptococcus*	0.00001795	0.0000813	Increased	0.0000	0.0002
*Intestinimonas*	0.00001079	0.0000861	Increased	0.0002	0.0014
*Senegalimassilia*	0.00001685	0.000108213	Increased	0.0002	0.0009
*Phocea*	0	0.0000934	Increased	0.0064	0.0255
*Conchiformibius*	0.00002235	0.000118051	Increased	0.0021	0.0095
*Erysipelatoclostridium*	0	0.000115167	Increased	0.0000	0.0000
*Anaerotruncus*	0.000009015	0.000143451	Increased	0.0000	0.0002
*Acinetobacter*	0.0000562	0.000205443	Increased	0.0002	0.0014
*Hungatella*	0	0.00017126	Increased	0.0000	0.0000
*Erwinia*	0.00001395	0.000199622	Increased	0.0000	0.0000
*Oscillibacter*	0.0000462	0.000247741	Increased	0.0000	0.0000
*Paraprevotella*	0.00003325	0.000311881	Increased	0.0000	0.0000
*Alistipes*	0.000326094	0.00097874	Increased	0.0001	0.0005
*Pseudomonas*	0.000324257	0.001701902	Increased	0.0000	0.0000
*Streptococcus*	0.000388367	0.00390552	Increased	0.0003	0.0017
*Tyzzerella*	0.0000317	0.005430396	Increased	0.0000	0.0000
*Solobacterium*	0.000758143	0.017759871	Increased	0.0000	0.0000
*Subdoligranulum*	0.003984826	0.022146933	Increased	0.0000	0.0000
*Phascolarctobacterium*	0.00381844	0.028108821	Increased	0.0022	0.0097
*Bacteroides*	0.011833503	0.046910755	Increased	0.0000	0.0000
*Fusobacterium*	0.000449843	0.066997124	Increased	0.0000	0.0000

## Data Availability

The raw sequencing data in FASTQ format is publicly available Zenodo data repository (https://doi.org/10.5281/zenodo.15663221), DOI: 10.5281/zenodo.15663221.
